# Box–Behnken Design for Assessing the Efficiency of Aflatoxin M1 Detoxification in Milk Using *Lactobacillus rhamnosus* and *Saccharomyces cerevisiae*

**DOI:** 10.3390/life13081667

**Published:** 2023-07-31

**Authors:** Mounir M. Salem-Bekhit, Omnia Karem M. Riad, Heba Mohammed Refat M. Selim, Sally Tohamy Kamal Tohamy, Ehab I. Taha, Saleh A. Al-Suwayeh, Gamal A. Shazly

**Affiliations:** 1 Kayyali Chair for Pharmaceutical Industries, Department of Pharmaceutics, College of Pharmacy, King Saud University, Riyadh 11451, Saudi Arabia; 2 Department of Microbiology and Immunology, Faculty of Pharmacy (Girls), Al-Azhar University, Cairo 11651, Egypt; dr_omniakarem@azhar.edu.eg (O.K.M.R.); dr_sallytohamy@azhar.edu.eg (S.T.K.T.); 3 Department of Pharmaceutical Sciences, Faculty of Pharmacy, Al-Maarefa University, Diriyah, Riyadh 13713, Saudi Arabia; 4 Department of Pharmaceutics, College of Pharmacy, King Saud University, Riyadh 11451, Saudi Arabia; eelbadawi@ksu.edu.sa (E.I.T.); ssuwayeh@ksu.edu.sa (S.A.A.-S.); gamalmym@gmail.com (G.A.S.)

**Keywords:** *L. rhamnosus*, *S. cerevisiae*, aflatoxin M1, animal milk, antiaflatoxigenic, Box–Behnken design

## Abstract

Milk contaminated with aflatoxin can lead to liver cancer. Aflatoxin B1 (AFB1), a serious animal feed contaminant, is transformed into Aflatoxin M1 (AFM1) and secreted in milk. In this study, a biological method using probiotic bacteria, *Lactobacillus rhamnosus* (*L. rhamnosus*) in combination with *Saccharomyces cerevisiae* (*S. cerevisiae*), was used to assess their antiaflatoxigenic effect in animal milk. A Box–Behnken design was used to establish the optimal ratio of *L. rhamnosus* and *S. cerevisiae*, incubation time, and temperature for efficient AFM1 detoxification from milk. To achieve this, the primary, interaction, and quadratic effects of the chosen factors were investigated. To investigate the quadratic response surfaces, a second-order polynomial model was built using a three-factor, three-level Box–Behnken design. The quantity of AFM1 was detected by the ELISA technique. The results of these experiments obtained an optimum condition in AFM1 detoxification of the three tested factors in order to maximize their effect on AFM1 detoxification in milk. The model was tested in three highly contaminated milk samples to assure the efficacy of the model. AFM1 detoxification was up to 98.4% in contaminated milk samples. These promising results provide a safe, low-cost, and low-time-consuming solution to get rid of the problem of milk contamination with AFM1.

## 1. Introduction

Aflatoxins are a collection of toxic substances secreted primarily by two fungal species, *Aspergillus flavus* and *Aspergillus parasiticus*, with over 20 types identified. The most common types of Aflatoxins are AFB1, AFB2, AFG1, AFG2, AFM1, and AFM2. AFM1 and AFM2 are produced from AFB1 and AFB2 metabolism, respectively [1]. Aflatoxins occur naturally in various foods and feedstuffs, including straws, forages, cornmeal, cottonseeds, almonds, and grains. The level of contamination may vary depending on the geographical region [2]. Fungal growth and subsequent aflatoxin production can be promoted by factors such as high temperatures, elevated humidity (80–90%), and plant injuries during processing and storage [3]. When cattle consume feed contaminated with AFB1, they excrete AFM1 through urine, milk, and feces, with the highest concentration in milk [4]. The transformation of AFB1 to AFM1 occurs in the liver through the action of cytochrome P450 [5]. The maximum level of AFM1 excretion in milk occurs within 48 h of consuming AFB-contaminated feed. The conversion of AFB1 to AFM1 can be influenced by dietary preferences, rates of ingestion and digestion, animal health, hepatic biotransformation capability, and milk production [6,7].

Aflatoxins pose a serious threat to the public’s health, because of their potent carcinogenic and mutagenic properties in humans [6]. Exposure to these toxins can cause encephalopathy, hepatic failure, Reye’s syndrome, and fetal and neonatal health and development issues [8]. About 25,200–155,000 new instances of hepatocellular carcinoma are thought to be caused by aflatoxin exposure each year worldwide [9]. AFB1, AFM1, and AFG1 have been classified as Group 1 carcinogens [10], which are chronic and acute poisons that can target several body organs, particularly the liver and kidneys. Exposure to aflatoxins is correlated with an increased risk of liver cancer, and the risk is increased in people infected with the hepatitis B virus (HBV). Additionally, prolonged aflatoxin exposure can cause immunosuppression, malnutrition, and birth abnormalities [11]. Many variables, such as nutritional condition, sex, age, exposure to viral hepatitis, and parasitic infection, can affect the severity of aflatoxin-induced diseases in humans [8].

Many nations have put in place stringent laws to avoid the toxicity of aflatoxins in food, including milk. For instance, the maximum residue level (MRL) of AFM1 in milk is 0.5 µg/L in the US, Brazil, and the majority of Asian nations, but only 0.05 µg/L in the majority of European nations [12]. Keeping the feed dry, which inhibits fungus growth and the creation of AFB1, is one of the greatest approaches to reducing AFM1 contamination in milk. Nevertheless, this is difficult in countries with hot and humid weather throughout the year. As an alternative control method, treating milk contaminated with AFM1 can be effective.

Numerous approaches were developed to limit AFM1 in milk, including physical, chemical, and biological control methods. Unfortunately, most of them are not widely used due to their high costs or practical difficulties [12]. Biological methods used include probiotic bacteria which are defined as live bacteria with benefits in the field of the food industry and human health. *Lactobacillus rhamnosus* is one of several probiotics commonly employed in the dairy product industry [13,14,15]. However, researchers are continuously exploring various strategies to manage aflatoxins in food. Various studies investigated the efficacy of different yeast strains, including *S. cerevisiae*, in binding AFM1 in milk [3]. To prevent fermentation, heat-killed cells have been used to preserve yeast cell membrane binding capacity and improve adsorption [16]. The binding process between AFM1 and living microorganisms is rapid, reaching maximum binding within minutes [17].

The interaction between microorganism cell membranes and aflatoxins is variable and may be attributed to the utilization of different strains [18]. Pierides and colleagues [19] found that the binding ability of microorganisms in phosphate buffer solution increased at acidic pH, but the precise interpretation remains unclear. To minimize bacterial fermentation during treatment, heat-killed bacteria were used to enhance AFM1 detoxification [16,20,21,22]. Regardless of the length of the treatment and the microbial strain, nonviable microorganisms’ ability to bind substances is more reliable than that of living ones [17,18,21]. Kuharić and colleagues [23] found that nonviable cells of *Lactobacillus plantarum* KM have more AFM1-detoxifying power than the viable cells. Although the precise process has not been fully understood, hydrogen bonds and van der Waals interactions are thought to be responsible for the weak link between aflatoxins and the microbe’s cell membrane. The adsorption of AFM1 is caused by polysaccharides and peptidoglycans, which are components of bacterial cell membranes [24]. Due to variations in cell membrane structure, different bacterial strains exhibit different adsorption abilities for aflatoxins [19].

It has been discovered that lactic acid bacteria (LAB) are efficient at binding AFM1 in tainted milk. The binding strength of LAB and aflatoxins is influenced by the used microbial strains, number of microorganisms, incubation time, and temperature.

The aim of this research was to optimize the detoxification of AFM1 from milk by investigating various factors. The selected factors were the ratio of *L. rhamnosus*:*S. cerevisiae* (X1), incubation temperature (X2), and incubation time (X3), while the observed variable (response) was the percentage detoxification (reduction) of AFM1 (Y). Response surface plots were used to depict the influence of the factors (X1), (X2), and (X3) on the response (Y) and to predict the optimal levels of factors X1, X2, and X3 for the highest reduction in AFM1 in milk samples.

## 2. Material and Methods

### 2.1. Chemicals and Media

Acetonitrile was supplied by S D Fine-Chem Ltd. (Mumbai, India). Other chemicals were purchased from Sigma-Aldrich (St. Louis, MO, USA). Regarding the culture media, de Man, Rogosa, and Sharpe (MRS) broth and agar were supplied from Oxoid Ltd. (UK). Also, Yeast extract peptone dextrose broth and agar were purchased from Oxoid Ltd. (UK).

### 2.2. Microbial Strains and Culture Conditions

*L. rhamnosus* (ATCC 7469) and *S. cerevisiae* (ATCC 24860) strains were obtained from the Biotechnology lab, Faculty of Pharmacy, King Saud University, KSU. To culture *L. rhamnosus*, begin by preparing the de Man, Rogosa, and Sharpe broth (MRS, Oxoid, UK) culture medium. A pure *L. rhamnosus* colony was inoculated and the culture was incubated at an appropriate temperature for 18 h under anaerobic conditions. For *S. cerevisiae*, Yeast extract peptone dextrose (Oxoid, UK) was used. A pure *S. cerevisiae* colony was inoculated in the medium and the culture was incubated at an appropriate temperature, usually between 28–30 °C for 24 h. To estimate the final bacterial count (10^7^ CFU/mL), dilution and plating procedures were used, and the suspension was stored at 4 °C till use.

### 2.3. Collection and Preparation of Milk Samples

Milk samples (10 raw milk samples) were gathered at random from several locations in Egypt’s Delta district. Sterile containers were used to collect the samples. The samples were stored at −20 °C until tested. 

Centrifugation and filtering were used as pretreatment processes to eliminate lipids and other contaminants that interfere with accurate AFM1 extraction and measurement. Acetonitrile (S.D. Fine-Chem Ltd., Mumbai, India) was used as an organic solvent for extraction. Preparation of milk samples was accomplished by placing 1 mL of milk samples in a 50 Ml centrifuge tube with 4 Ml of acetonitrile, and the mixture was allowed to stand for five minutes before being centrifuged at 4000 rpm for ten minutes at 25 °C. A total of 2.5 mL of supernatant was dried in a water bath. Tubes were kept dried until use, and 1 mL of the redissolving solution from the ELISA kit was added before further analysis and quantification of AFM1. 

### 2.4. Use of Box–Behnken Design

A statistical modeling method known as the Box–Behnken design of experiments (BBD) enables the identification of significant variables in a given field with a constrained number of trials. In order to achieve the ideal state, it also enables the prediction of the best levels of variables [25]. In the current study, milk samples with higher AFM1 contamination (MS-T02, MS-H04 and MS-M09) were selected for evaluating the effect of *L. rhamnosus*:*S. cerevisiae* ratio, incubation temperature, and incubation time (independent variables) on the percent reduction in AFM1 (the dependent variable). To examine the effect of different levels of independent variables on the dependent variable, 15 runs—obtained from the Box–Behnken design—had been used, according to Table 1 and Table 2. 

### 2.5. Assay of AFM1

The quantity of AFM1 was detected using the Enzyme-Linked Immune Sorbent Assay (ELISA) method with the AFM1 test kit (Cat. no. E4566, Biovision, CA, USA). The kit was stored at 8 °C and left for one hour before use. Standard solutions and the prepared samples were placed in separate wells of 96 microtiter plate and kept at room temperature in a dark place for one hour. After incubation, the liquid was removed, and the wells were washed twice. Enzyme conjugate was added and incubated, followed by washing and the addition of substrate and chromogen solutions. The wells were incubated in a dark place for 30 min and the stop reagent was added and mixed. With the ELISA reader, the absorbance of AFM1 was measured photometrically at 450 nm against an air blank. The concentration of AFM1 in the milk samples was calculated by comparing the absorbance percentages to a calibration curve that was created using standards at various concentrations.

## 3. Results and Discussion

Food control must be given a lot of effort, as food safety and foodborne diseases are considered major growing health problems. Milk is a significant source of the human diet because it offers a natural, high-quality source of bioavailable calcium and proteins; thus, human health at different ages is strongly influenced by the quality of milk products. Unfortunately, milk content supports the growth of different pathogens, and also could be contaminated with other toxins during transfer. Many researchers have confirmed the presence of high concentrations of mycotoxins in milk [26,27]. The presence of mold contamination not only degrades the quality of food and animal feed, but it can also have adverse effects on human health. The potential health risks associated with AFM1 exposure have been widely documented in previous studies [28,29]. High levels of AFM1 exposure were associated with an increased risk of liver cancer [30]. AFM1 contamination of milk has projected ramifications that by 2030, the maximum mean of AFM1 in milk will have increased by up to 50% [31].

**Figure 1:**
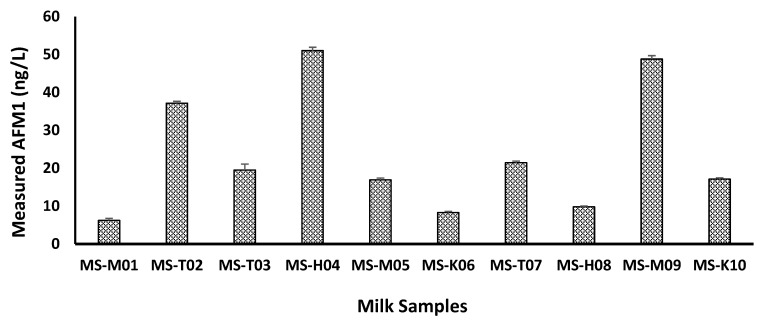
The levels of AFM1 (ng/L) in ten tested raw milk samples.

AFM1 is a toxic metabolite secreted by certain strains of *Aspergillus fungi*, and can cause health hazards to humans. Through the utilization of the competitive ELISA technique, the study analyzed ten raw milk samples to decide the occurrence and levels of AFM1 contamination. Figure 1 displays the results of the analysis, which revealed that all of the investigated raw milk samples were contaminated with AFM1. The lowest AFM1 contamination levels were 6.2 ± 0.55, 8.30 ± 0.33, and 9.80 ± 0.24 ng/L, while the highest concentrations were 37.12 ± 0.51, 48.80 ± 0.90, and 51.0 ± 0.92 ng/L.

The ELISA technique is widely utilized for the detection of aflatoxin M1 (AFM1), a potent mycotoxin commonly found in milk and dairy products. However, it is crucial to acknowledge the inherent limitations of ELISA in AFM1 detection, highlighting the need for confirmatory analysis using liquid chromatography–mass spectrometry/mass spectrometry (LC-MS/MS) in future research or studies conducted by other laboratories. This approach provides a more precise and dependable assessment of AFM1 levels in food samples, ensuring the safety and quality of dairy products without duplicating previous work.

Previous research has also documented the occurrence of AFM1 contamination in dairy products, including raw milk [28]. Our study was comparable to research performed by Busman et al. [32] who stated that 24 out of 26 raw milk samples were contaminated with AFM1. Similarly, a study in Pakistan by Hussain and Anwar [33] reported the occurrence of AFM1 in all of the analyzed raw milk samples, with the highest concentration at 0.57 µg/L. Additionally, a prior study in Egypt found that raw milk samples taken in a two-year period (2016–2017) were contaminated with AFM1 at rates of 21.6% and 18.3%, respectively, exceeding the legal European limit (0.05 µg/L) by 100% and 90.9% [34]. This difference may be due to some factors explained by Ismaiel and his colleagues, who attributed fluctuation in aflatoxin contamination levels to variations in practices of feed storage and farm management [34]. In addition to that another study by Iqbal and his colleagues stated that geographical conditions, climate, seasonal variations, various toxin detection methods, and farm management practices are all factors that could lead to these fluctuations in AFM1 concentration in milk. Changes in temperature and the occurrence of severe weather events, such as droughts and floods, can have an indirect impact on milk production and its quality [12]. This is mainly due to the alteration in the accessibility and quality of feed and water resources. [35]. In addition to that, the vast variance in aflatoxin M1 contamination in milk was linked to many parameters; season, animal type, milking time, AFB1 intake, and amount of produced milk [3,34]. 

Effective measures to prevent and minimize AFM1 contamination in dairy products have been extensively studied. Several strategies, including chemical, physical, and biological methods, were studied to eliminate AFM1 [36]. The research performed by Khoori and coworkers investigated the effectiveness of various dairy processing techniques, including ultraviolet radiation, ozonation, and pulsed electric field processes, in controlling *Aspergillus* species in animal food [37].

Recently, biological methods have shown promise in reducing the level of AFM1 in raw milk. It is well recognized that biological detoxification is extremely efficient and has benefits such as ecological sustainability, applicability over a variety of mycotoxins, ease of use, and cost-effectiveness [38]. The most successful and promising methods for removing AFM1 from milk and dairy products involve adsorption through nonviable bacteria and clay materials. [36]. Biological detoxification can be carried out by different microorganisms such as bacteria, fungi, or algae. Algae and the molecules they create have a variety of qualities, including those that are antifungal, antioxidant, antibiofilm, and many more. These features can be used for a variety of purposes, including the detoxification or breakdown of harmful substances, such as mycotoxins. Sulfated polysaccharides, β-D-glucans, polyphenolic compounds, etc., are some examples of these substances. One of these algae, Spirulina platensis, has been proposed as a potential aflatoxin detoxicant [38]. Bacteria such as *Lactobacillus* and *Bifidobacterium* have been found to have the power to reduce AFM1 in raw milk [39]. These bacteria are capable of producing enzymes that can degrade the toxin, thereby reducing its concentration in the milk [40]. In addition, these bacteria can also modify the pH of the milk, which can further aid in reducing the toxicity of AFM1 [41]. 

**Figure 2:**
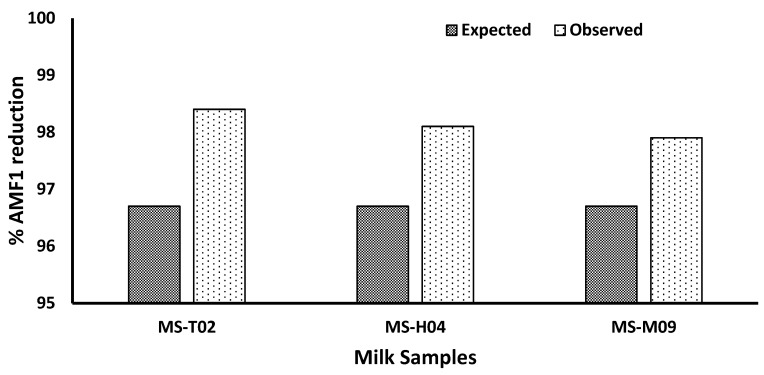
Expected and observed percent AFM1 reduction values (*p* ≤ 0.05).

In the current study, probiotic bacteria, *L. rhamnosus* blended with *S. cerevisiae*, were used to evaluate their deaflatoxigenic effect in raw milk. We investigated the effect of three factors that we believe have a high impact on the detoxification of AFM1 from a raw milk sample. These factors are *L. rhamnosus*:*S. cerevisiae* (LR:SC) ratio, incubation temperature (°C), and incubation time (min.) as shown in Table 1. According to the Box–Behnken design, three levels from each factor were used to create 15 experimental runs, as seen in Table 2. These factors were tested in the three highly contaminated milk samples (MS-T02, MS-H04, MS-M09) with concentrations of 37.12 ± 0.51, 51.0 ± 0.92, and 48.80 ± 0.90 (ng/L), respectively. At LR:SC ratio = 1.6:1, incubation temperature 47 °C, and incubation time = 79 min, the observed percent reduction in AMF1 for samples (MS-T02, MS-H04, MS-M09) were 98.4, 98.1, and 97.9%, respectively (Figure 2). Accordingly, the optimization design created the following quadratic equation in terms of the studied factors

AFM1 5 reduction = 95.7 + 4.12X_1_ + 2.12X_2_ + 0.5X_3_ − 1.75 X_1_X_2_ − 7.71 X_1_^2^ − 1.71 X_2_^2^ − 0.46 X_3_^2^

where X_1_ is the LR:SC ratio, X_2_ is the temperature, X_3_ = time.

This equation is a useful tool for predicting the reaction for different values of each element. By comparing the factor coefficients, it can also help identify the relative impact of the variables.

At LR:SC ratio = 1.6:1, incubation temperature 47 °C, and incubation time = 79 min (Table 3), the observed percent reduction in AMF1 for samples (MS-T02, MS-H04, MS-M09) were 98.4, 98.1, and 97.9%, respectively (Figure 2).

Table 4 presents the analysis of variance (ANOVA) of the quadratic model with regard to the percent reduction in AMF1. The significant model F-value of 20.90 suggests that the model is significant, and there is a low chance (0.19%) that such a large F-value could occur due to noise. Significant model values have *p*-values less than 0.05. In this case, X1, X2, and X12 are significant model terms. Model terms with values higher than 0.1 are considered insignificant.

**Figure 3:**
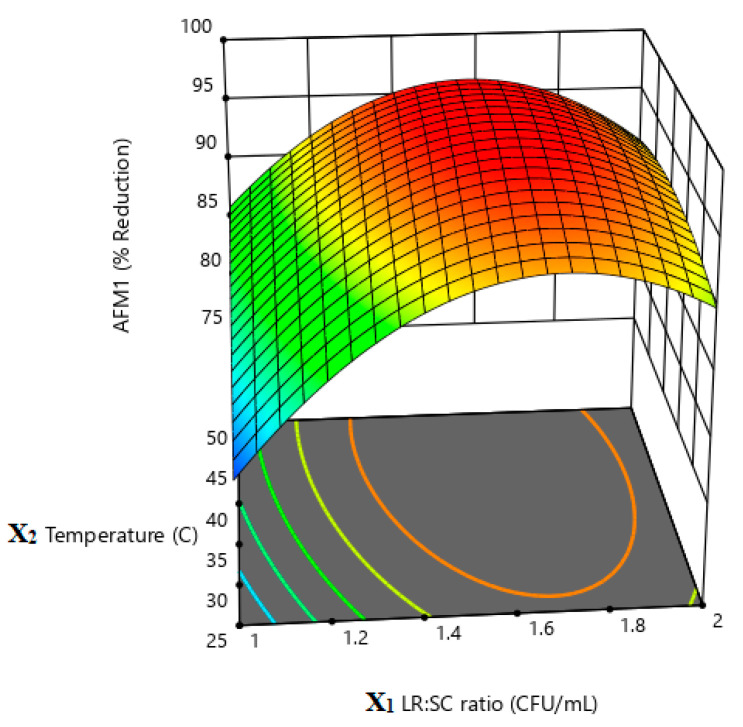
Response surface plot illustrates the effect of LR:SC ratio (X1) and incubation temperature (X2) on % AFM1 reduction.

**Figure 4:**
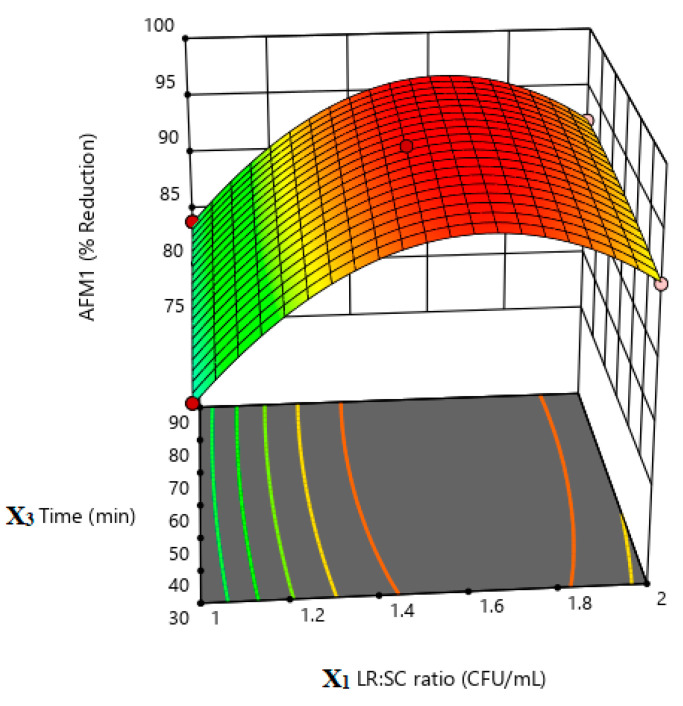
Response surface plot illustrates the effect of LR:SC ratio (X1) and incubation time (X3) on % AFM1 reduction.

**Figure 5:**
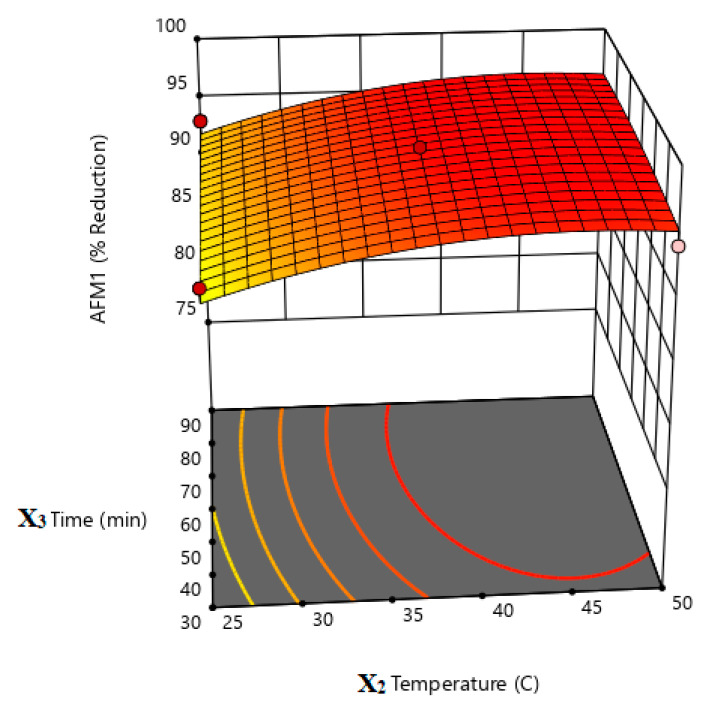
Response surface plot illustrates the effect of incubation temperature (X2) and incubation time (X3) on % AFM1 reduction.

According to the obtained quadratic equation and the results of the response surface plots (Figure 3, Figure 4 and Figure 5), it is obvious that X1 (*L. rhamnosus*:*S. cerevisiae* ratio) and X2 (incubation temperature) have the highest impact on the percent reduction in AMF1 in milk samples. This could be concluded from the coefficient of each term in the given equation. The higher the coefficient, the higher the impact of this factor in the measured response, and vis versa (Table 5). The positive and negative sign of the coefficient indicate a direct or inverse effect of this factor on the response, respectively [42].

The optimized factor levels shown in Table 3 were tested in three highly contaminated milk samples (MS-T02, MS-H04, MS-M09) with concentrations of 37.12 ± 0.51, 51.0 ± 0.92, and 48.80 ± 0.90 (ng/L), respectively, and the resulting percent AFM1 reduction was in close agreement with the predicted value. 

The antiaflatoxigenic effect of *L. rhamnosus* and *S. cerevisiae* is documented in several studies demonstrating the physical binding of these microorganisms to aflatoxins [43,44]. *Lactobacillus rhamnosus* and *Saccharomyces cerevisiae* cell walls immobilized on nanosilica trapped in alginate were used by Vahidimehr and his coworkers as aflatoxin M1 (AFM1) binders. Following that, they were in contact with AFM1 for 15 and 24 h. For the free cell wall combination at 15 min, the results demonstrated an AFM1 reduction ranging from 53 to 87% [43]. In another investigation, the adsorption capacities of yeast (*Saccharomyces cerevisiae* ), activated charcoal, and the probiotic *Lactobacillus rhamnosus* as AF adsorbents were evaluated. They reported the highest adsorption efficiency (96.8%) with this combination treatment [44]. Ismail et al. [16] evaluated the potential of *S. cerevisiae* to bind AFM1 at different levels and reported that 100% of AFM1 (0.05 µg/L) in spiked artificial milk was reduced to trace concentrations by *S. cerevisiae* alone or in combination with LAB. At 0.1 µg/L, *S. cerevisiae* at a concentration of 10^10^ CFU/mL reduced AFM1 in milk by 92%. Abdelmotilib and colleagues demonstrated that a combination of LAB and *S. cerevisiae* could degrade up to 90% of AFM1 in milk with an initial concentration of 50 ng/mL [45]. Additionally, Gu et al. demonstrated that Bacillus supernatant was able to reduce AF by 76.9% in a solvent [46]. However, a single strain of LAB was found to be less effective, resulting in less than a 90% reduction in AFM1 in milk. Probiotic bacteria and yeasts were also observed to decrease AFM1 contamination in milk by 19–61%, particularly at a high contamination level of 50 ng/mL [47]. In a similar study, two types of lactobacilli (*L. rhamnosus*, *L. plantarum*) were successfully used with *S. boulardii* to detoxify AFM1 in animal milk by 97.1–100%. At doses of 107 and 109 (CFU/mL), *L. rhamnosus* showed the highest binding capacity, removing 82% and 90% of the AFM1 from milk samples containing 0.5 and 0.75 ng/mL, respectively. A mixture of the three mentioned probiotic bacteria was used at a concentration of 1 × 10^7^ CFU/mL at 37 °C with 0.5 ng/mL AFM1, and this mixture produced the highest AFM1 binding (100.0 ± 0.58) [48].

These results reveal the potential risks of AFM1 contamination in raw milk. As a toxic metabolite, AFM1 not only degrades the quality of milk but also poses a serious threat to human health. Correspondingly, these findings are concerning, as consuming raw milk contaminated with AFM1 can lead to various health complications, including liver damage and an increased risk of liver cancer. Therefore, it is crucial to monitor and regulate the presence of this toxin in raw milk to ensure public health and safety.

Our research outcomes hold great potential in contributing to the development of enhanced strategies for AFM1 detoxification in milk, utilizing the selected probiotic bacteria, *Lactobacillus rhamnosus* and *Saccharomyces cerevisiae*. By thoroughly investigating the detoxification mechanisms of these strains and optimizing their effectiveness, our research can provide valuable insights for the food industry. The implementation of these probiotics as a detoxification approach has the capacity to significantly improve food safety by reducing AFM1 levels in milk, thereby reducing the health risks associated with AFM1 contamination. This natural and sustainable alternative to chemical methods offers the advantage of ensuring consumer confidence in the quality and safety of dairy products. Ultimately, our research has the potential to shape improved practices within the food industry, leading to the production of safer milk products and protecting public health.

## 4. Conclusions

This study emphasizes the importance of continuously monitoring and managing AFM1 levels in raw milk and dairy products. Ensuring effective measures to minimize contamination and exposure to AFM1 is critical in maintaining public health and safety. Box–Behnken design is a very useful tool for saving time and effort to optimize the condition and process of experiments. In our study, factors affecting AFM1 detoxification from milk were optimized in order to maximize the efficiency of the detoxification process. In this investigation, AFM1 detoxification was up to 98.4% in contaminated milk samples. Other conditions such as the type of *Lactobacillus bacteria* and yeast, as well as other ratios, need to be optimized as well to maximize the detoxification process. 

## Figures and Tables

**Table 1:** Variables (dependent and independent) in the Box–Behnken design.

Independent Variables	Levels		
	Low	Medium	High
(X1) LR:SC ratio	1:1	1.5:1	2:1
(X2) Incubation temperature (°C)	25	37.5	50
(X3) Incubation time (min.)	30	60	90

LR, *L. rhamnosus*; SC, *S. cerevisiae*.

**Table 2:** Box–Behnken runs for both factors and the corresponding results of AFM1 % reduction.

Run	LR:SC Ratio	Temperature (°C)	Time (min)	AFM1 % Reduction (Y)
1	2	37.5	90	92
2	1.5	37.5	60	95
3	1.5	25	30	92
4	1.5	25	90	93
5	1	37.5	90	84
6	2	37.5	30	91
7	1	25	60	77
8	1.5	50	30	94
9	1.5	37.5	60	96
10	2	25	60	89
11	1	37.5	30	83
12	1.5	37.5	60	96
13	2	50	60	92
14	1	50	60	87
15	1.5	50	90	95

LR, *L. rhamnosus*; SC, *S. cerevisiae*. AFM1 was quantified in the collected milk samples using ELISA technique.

**Table 3:** Optimized levels of values factors in Box–Behnken design.

Factor	Optimized Levels of Factors
LR:SC ratio	1.6:1
Incubation temperature (°C)	47
Incubation time (min)	79

**Table 4:** ANOVA for quadratic model: response (AFM1).

Source	Sum of Squares	df *	Mean Square	F-Value	*p*-Value
X1 (LR:SC ratio)	136.12	1	136.12	62.35	0.0005
X2 (Temperature)	36.12	1	36.12	16.55	0.0097
X3 (Time)	2.00	1	2.00	0.9160	0.3825
X1X2	12.25	1	12.25	5.61	0.0641
X1X3	0.00	1	0.00	0.00	1.0000
X2X3	0.00	1	0.00	0.00	1.0000
X1	219.39	1	219.39	100.48	0.0002
X2	10.78	1	10.78	4.94	0.0770
X3	0.7756	1	0.7756	0.3553	0.5771
Model	410.7	9	45.63	20.90	0.0019

ANOVA: Analysis of variance; * df: degree of freedom.

**Table 5:** Coefficients in terms of factors.

Factor	Coefficient Estimate	df *	Standard Error	95% CI * Low	95% CI High
Intercept	95.7	1	0.8531	93.47	97.86
X_1_ (LR:SC ratio)	4.12	1	0.5224	2.78	5.47
X_2_ (Temperature)	2.12	1	0.5224	0.7821	3.47
X_3_ (Time)	0.50	1	0.5224	−0.8429	1.84
X_1_X_2_	−1.75	1	0.7388	−3.65	0.1492
X_1_X_3_	0.00	1	0.7388	−1.90	1.90
X_2_X_3_	0.00	1	0.7388	−1.90	1.90
X_1_^2^	−7.71	1	0.7690	−9.69	−5.73
X_2_^2^	−1.71	1	0.7690	−3.69	0.2684
X_3_^2^	−0.46	1	0.7690	−2.44	1.52

* df is the degree of freedom, * CI is the confidence interval.

## Data Availability

Data are contained within the article or are available from the authors upon reasonable request.
